# Supplementary material for: Effect of Drug Loading in Mesoporous Silica on Amorphous Stability and Performance
Source: Pharmaceutics. 2024 Jan 24;16(2):163. doi: 10.3390/pharmaceutics16020163 (PMC10891643; doi:10.3390/pharmaceutics16020163)
Supplement: Supplementary file 1 [file pharmaceutics-16-00163-s001.zip › pharmaceutics-2768862-supplementary.pdf]

Supplementary material

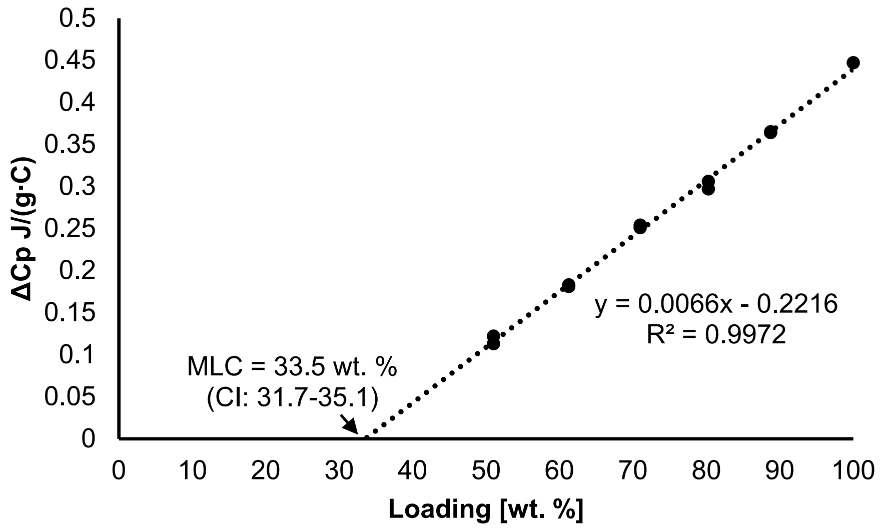

Figure S1 Experimental monomolecular loading capacity (xMLC)

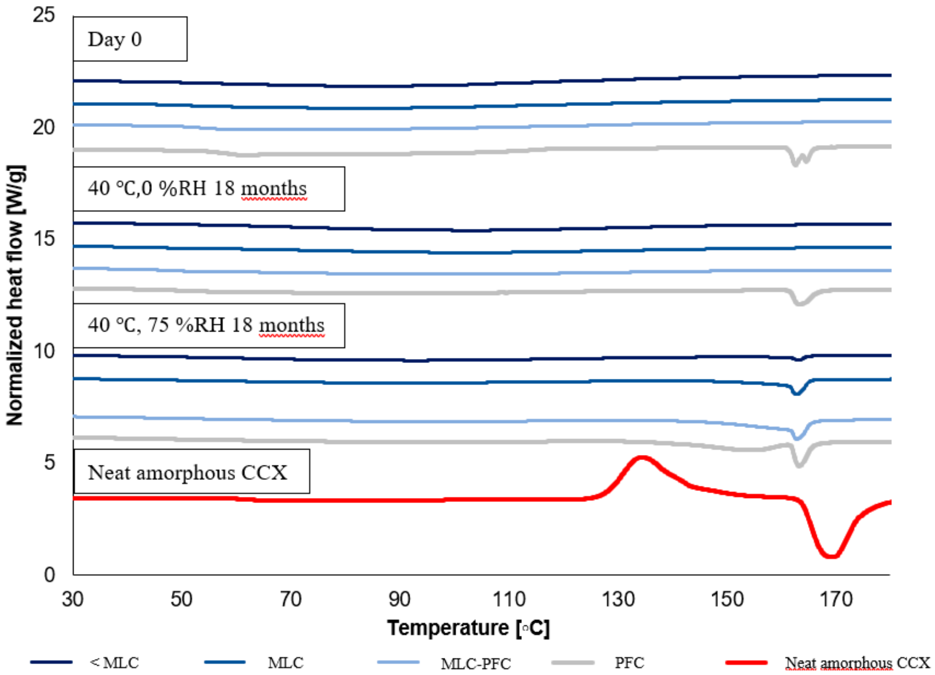

Figure S2 Differential scanning calometry themrograms for the freshly prepared samples, the same sanples after 18 months storage at 40°C/ 0%RH and 40°C/75%RH as well as the crystalline CCX reference.

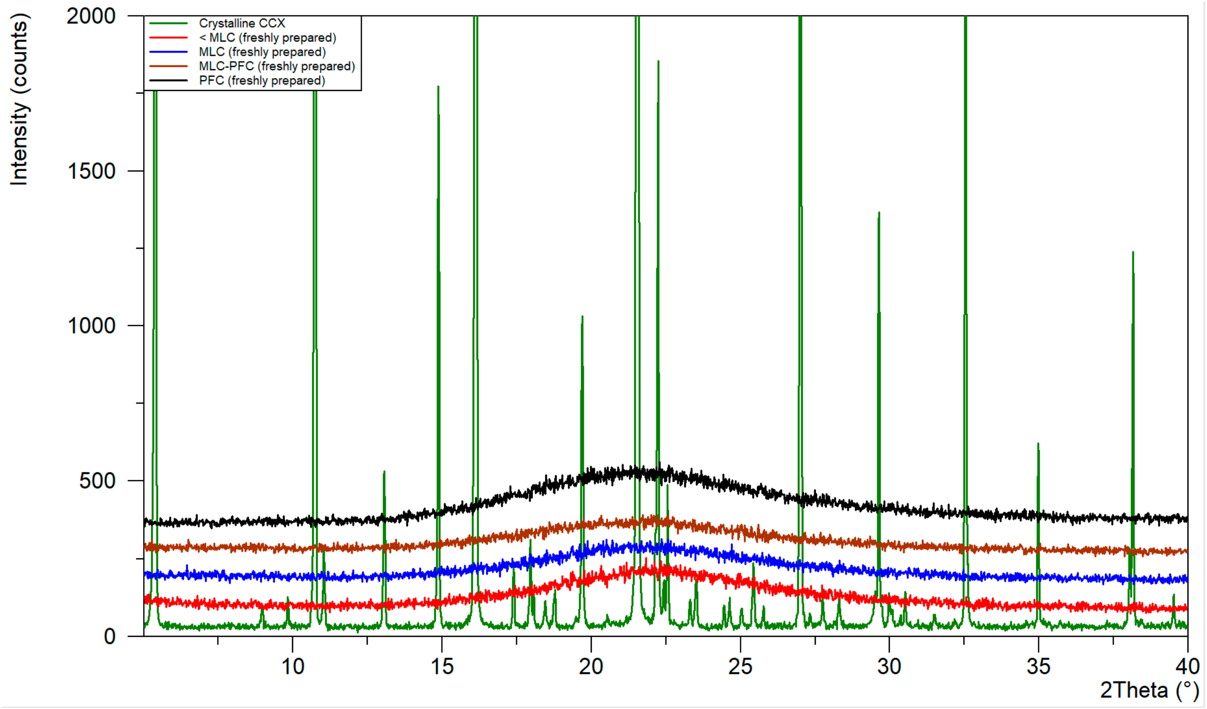

Figure S3 X-ray diffractograms of the freshly prepared samples as well as the crystalline CCX reference.

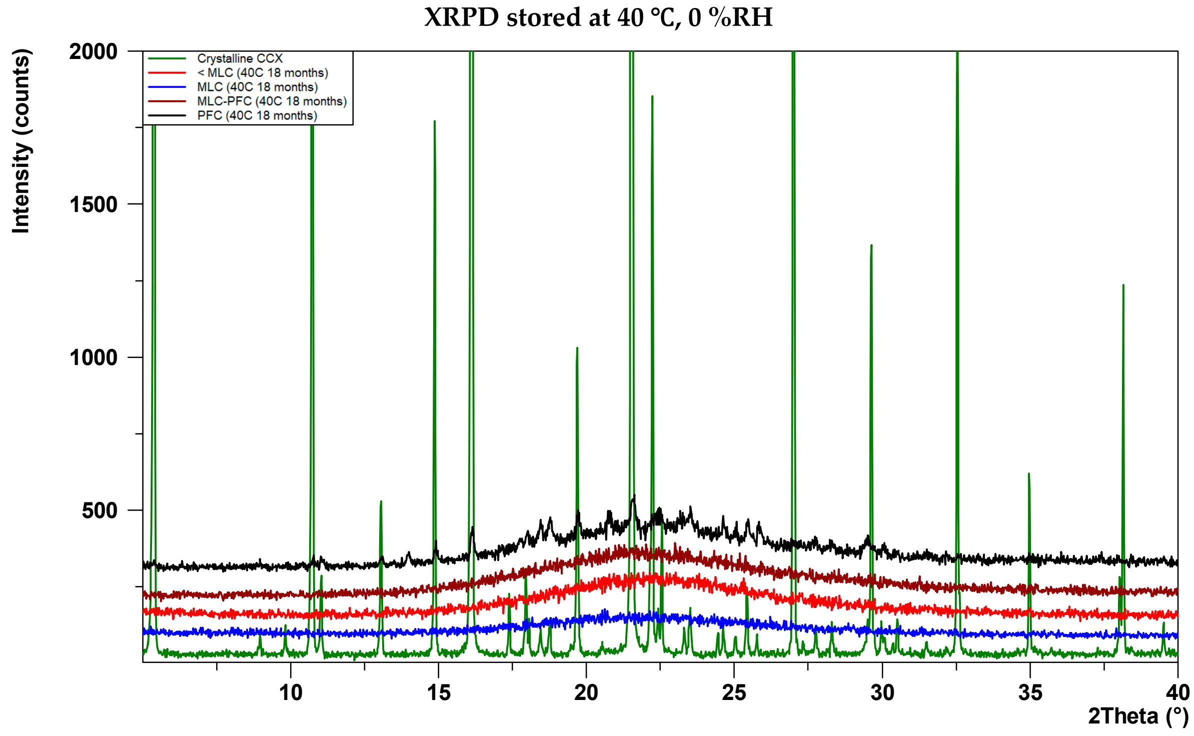

Figure S4 X-ray diffractograms of the samples stored for 18 months at 40 ℃, 0 %RH as well as the crystalline CCX reference.

XRPD stored at 40 ℃, 75 %RH

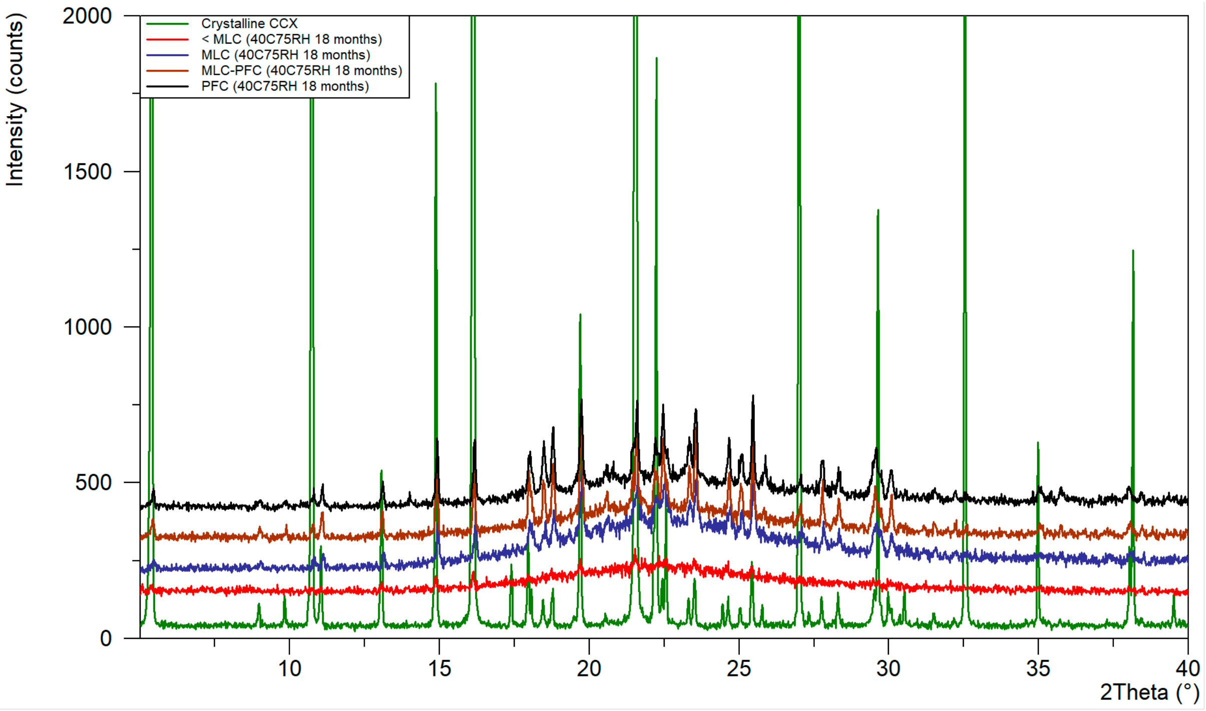

Figure S5 X-ray diffractograms of the samples stored for 18 months at 40 ℃, 75 %RH as well as the crystalline CCX reference.
